# Identification of Potential Herbal Inhibitor of Acetylcholinesterase Associated Alzheimer's Disorders Using Molecular Docking and Molecular Dynamics Simulation

**DOI:** 10.1155/2014/705451

**Published:** 2014-05-14

**Authors:** Chandrabhan Seniya, Ghulam Jilani Khan, Kuldeep Uchadia

**Affiliations:** ^1^Department of Biotechnology, Madhav Institute of Technology and Science, Race Course Road, Gola Ka Mandir, Gwalior 474005, India; ^2^School of Engineering, The University of Warwick, Coventry CV4 7AL, UK; ^3^Profarm Seed India Pvt. Ltd, 9 Anthem, Gundala Pochampally Village, Secunderabad, Andhra Pradesh 500014, India

## Abstract

Cholinesterase inhibitors (ChE-Is) are the standard for the therapy of AD associated disorders and are the only class of approved drugs by the Food and Drug Administration (FDA). Additionally, acetylcholinesterase (AChE) is the target for many Alzheimer's dementia drugs which block the function of AChE but have some side effects. Therefore, in this paper, an attempt was made to elucidate cholinesterase inhibition potential of secondary metabolite from *Cannabis* plant which has negligible or no side effect. Molecular docking of 500 herbal compounds, against AChE, was performed using Autodock 4.2 as per the standard protocols. Molecular dynamics simulations have also been carried out to check stability of binding complex in water for 1000 ps. Our molecular docking and simulation have predicted high binding affinity of secondary metabolite (C_28_H_34_N_2_O_6_) to AChE. Further, molecular dynamics simulations for 1000 ps suggest that ligand interaction with the residues Asp72, Tyr70-121-334, and Phe288 of AChE, all of which fall under active site/subsite or binding pocket, might be critical for the inhibitory activity of AChE. This approach might be helpful to understand the selectivity of the given drug molecule in the treatment of Alzheimer's disease. The study provides evidence for consideration of C_28_H_34_N_2_O_6_ as a valuable small ligand molecule in treatment and prevention of AD associated disorders and further *in vitro* and *in vivo* investigations may prove its therapeutic potential.

## 1. Introduction

The loss of neurons in the central nervous system and nerve cell dysfunction that lead to neurodegenerative disorder was first discovered in 1907 by a German scientist, Alois Alzheimer, and named as Alzheimer's disease (AD). Alzheimer's disease and other age-related memory disorders always remain key interest of researchers worldwide. AD is a slowly progressive disorder and is characterized by appearance of neurofibrillary tangles, neuritic plaques, rapid loss of synapses, and degeneration of basal cholinergic neurons [1] and disturbances in reasoning, planning, perception, and rational thinking [2, 3]. The number of individuals with AD in the USA is expected to be 14 million by the year of 2050 [4]. Hence, there is a need for development of novel treatments to reduce the risk.

Acetylcholinesterase (EC 3.1.1.7), also known as AChE, is the most essential enzymes in the family of serine hydrolases, which plays a key role in memory and cognition [5, 6]. Cholinesterase is the only target that has resulted in the design of a few calming drugs presently marketed for the treatment of AD [7]. Recent reports on therapeutic approaches to AD disease are based on the assumption of a cholinergic mechanism, with particular emphasis on AChE inhibition [8]. Undeniably, many scientific trials have been conducted in order to discover emblematic drug for the treatment of AD. So far, only seven drugs Caproctamine, Donepezil, Galanthamine, Huperzine, Memantine, Rivastigmine, and Tacrine [9, 10] have been approved by the Food and Drug Administration (FDA or USFDA) for the treatment of AD. Additionally, due to numerous side effects such as hepatotoxicity, gastrointestinal disturbance, dizziness, diarrhea, vomiting, nausea, pharmacokinetic disadvantages [11], and limited number of therapeutic options for AD, there is a call to discover new more effective compounds.

The natural compounds have always been served as a useful source to study inhibitory effect on AChE activity. A number of phytochemicals, namely, alkaloids, pregnane glycosides (cynanchoides), stilbenes, triterpenes [12], ursane [13], and xanthones, have shown AChE inhibitory activity. However, tetrahydrocannabinol (THC) is a pharmacologically active secondary metabolite in* Cannabis *plant and is one of the oldest hallucinogenic drugs and found to inhibit AChE more effectively than commercially marketed drugs [14, 15].

The development of new drugs is needed to persuade bountiful clinical applications in abundant diseases implications. Nowadays, scientists are currently producing a large set of 3D structure data and number of therapeutic lead compounds as well. Hence, to deal with such a large set of data and to design new therapeutic compounds, drug discovery process requires virtual screening of drugs, molecular docking and molecular dynamics simulations studies.

Virtual screening along with molecular modeling and docking is helping to design active novel enzyme inhibitors and pharmacophores that bind receptor sites. In this study, we have identified both qualitative and quantitative pharmacophore natural molecules based on AChE inhibition collected from the ChemBank database. The results were compared and the best inhibitor lead compounds were identified. The potential hit compounds obtained from this study can be further evaluated by* in vitro* and* in vivo* biological tests.

## 2. Materials and Methods

### 2.1. Protein Selection and Preparation

PDB structure of unliganded AChE (pdb id: 2W9I) was retrieved from RCSB Protein Data Bank (PDB) (http://www.rcsb.org/). The protein prepared for molecular docking by removal of water molecules, metal ions, and cofactors and addition of charges and hydrogen atoms using SPDBV (http://spdbv.vital-it.ch/) and energy minimization of 3D structures was done by using Yet Another Scientific Artificial Reality Application (YASARA) [16]. To depict the* in vivo *interaction, the energy was minimized of the target protein before performing the docking and molecular dynamics simulations.

### 2.2. Structural Features of Acetylcholinesterase

The biochemical and biological activity of* Acetylcholinesterase *depends on the hydrophobic active site which could be divided into several subsites such as oxyanion hole (Gly121, Gly122, and Ala204), anionic subsite (Trp86, Tyr133, Glu202, Gly448, and Ile451), and acyl binding pocket (Trp236, Phe295, Phe297, and Phe338). The catalytic triad (Ser203, His447, and Glu334) is located in the active site of the narrow deep gorge [17]. The peripheral anionic site (PAS) comprising set of aromatic residues (Tyr72, Asp74, Tyr124, Ser125, Trp286, Tyr337, and Tyr341) is located at the rim of the gorge and provides a binding site for allosteric modulators, inhibitors, and other residues of the omega group (Thr83, Asn87, and Pro88) [18]. The omega group residues form a disulphide linked loop (Cys69-Cys96), located at the bottom in the centre of the molecule and cover the enzyme's active site [19]. The residue Trp286 plays a very important role in ligand binding in the PAS. Ligand binding to the PAS affects/inhibits enzymatic activity through a combination of steric blockade of ligands moving through the gorge and by allosteric alteration of the catalytic triad conformation and efficiency [20, 21].

### 2.3. Virtual Screening and Docking of Ligands

AutoDock4.2 suite was used as molecular docking tool in order to carry out the docking simulations [22, 23]. Recently, molecular docking of pharmacophores to 3D models of a protein is a good choice for drug discovery process. The wide range of tetrahydrocannabinol derivatives (Δ-9-THC) in the training set allowed for the screening of large ChemBank database (http://chembank.broadinstitute.org/chemistry/search/input/userList.htm). The ligands were virtually screened on the basis of Lipinski's “rule of five” that sets the criteria for drug-like properties [24]. Structures were drawn using ACDLABS (http://www.acdlabs.com/) and converted into PDB coordinate files by using OPENBABEL software. H-atoms were added to the target protein for correct ionization and tautomeric states of amino acid residues and the nonpolar hydrogens were then merged as well. Kollman united atom charges and solvation parameters were assigned to the proteins and the Gasteiger charge was assigned to the ligand. The modified structures obtained were converted to PDBQT format in ADT for AutoDock calculations. The Lamarckian Genetic Algorithm was implemented with a population size of 150 dockings and 2.5 million energy evaluations for all docking experiments. All other parameters were run with default settings such as crossover rate and mutation rate. The grid size for specifying the search space was set at 60 × 60 × 60 centered on the macromolecule with a default grid point spacing of 0.375 Å. Precalculated grid maps were obtained using AutoGrid, which store grids of interaction energy based on the interaction of the ligand atom probes with receptor target. The similarity conformations of docked structures were measured by computing root mean square deviations between the coordinates of the atoms and creating clustering of the conformations based on the RMSD values and the lowest binding energy conformation in all clusters was considered the most favorable docking pose (Figure 3).

### 2.4. Analysis and Confirmation of Docking Results

The outputs from AutoDock and molecular dynamics simulation studies as well as images were generated with PyMol [25]. Docking logs were analyzed in the graphical user interface of Auto Dock Tools (ADT) and Python scripts in MGL tools package were used to analyze the docking results [26]. Hydrogen bonds lengths were measured with its binding partner using Ligplot software (http://www.ebi.ac.uk/thornton-srv/software/LIGPLOT/).

### 2.5. MD Simulations in Water

The Gromacs4.5.5 package (http://www.gromacs.org/) was used to prepare the protein and the ligand files as well as for the Molecular Dynamics (MD) simulations. GROMACS is a high-end, high performance research tool designed for the study of protein dynamics using classical molecular dynamics theory [27, 28]. The binding complex of AChE/CID1990283 (C_28_H_34_N_2_O_6_) obtained using AutoDock4.2 was simulated in neutral condition by adding appropriate number of sodium counter-ions and was solvated in an octahedron box of SPC/E water model [29] with a 1.0 Å distance between the protein surface and the box boundary [30]. Under coulombtype, PME stands for “Particle Mesh Ewald” electrostatics [31]. PME was the best method for computing long-range electrostatics (gives more reliable energy estimates especially for systems where counter ions like Na^+^, Cl^−^, Ca^2+^, etc. are used). It was even more beneficial for us to use counter ions to balance the charge and set the system to net neutral, if not, PME will not give reliable results. For fixing all bond lengths in the system the Linear Constraint algorithm has been used by using the all-bonds option under constraints (important to use this option when *dt* > 0.001 ps) [32]. The system was equilibrated beginning with the protein atom restrained simulations having 100 ps equilibration dynamics of the solvent molecules at 300 K. The equilibration of the solute molecules with a fixed configuration of the solvent molecules was the next step involved in which the system was slowly heated from temperature 50 to 300 K in 60 intervals each involving heating for a 5 K increase in 2.5 ps followed by an equal time equilibration. The entire system was equilibrated at 300 K for 100  ps before a sufficiently long MD simulation (for 1000 ps) at room temperature. The MD simulations were performed with a periodic boundary condition in the NPT ensemble at temperature of 5 K to 298.15 K with Berendsen temperature coupling and constant pressure *P* 51 atm with isotropic molecule-based scaling. We used a time step of 1 ps and a nonbond interaction cutoff radius of 10 Å. MD simulations were performed on Ubuntu11.0 Linux operating system at the Department of Biotechnology, Madhav Institute of Technology and Science, Gwalior.

## 3. Results and Discussion

The molecular alignment is done according to the electrostatic and structural properties of the active site of AChE. The steric, electrostatic, and hydrophobic fields were mapped onto the active binding pocket of AChE to better understand AChE and ligand interactions. Early inhibition research was mainly focused on ligands binding in the active site. The recent efforts have focused on finding novel ligands that bind to both sites in order to search for more potent reversible inhibitors. Prediction of interactions between small molecules and proteins is a crucial step to decipher many biological processes and plays a critical role in drug discovery.

**Figure 1:**
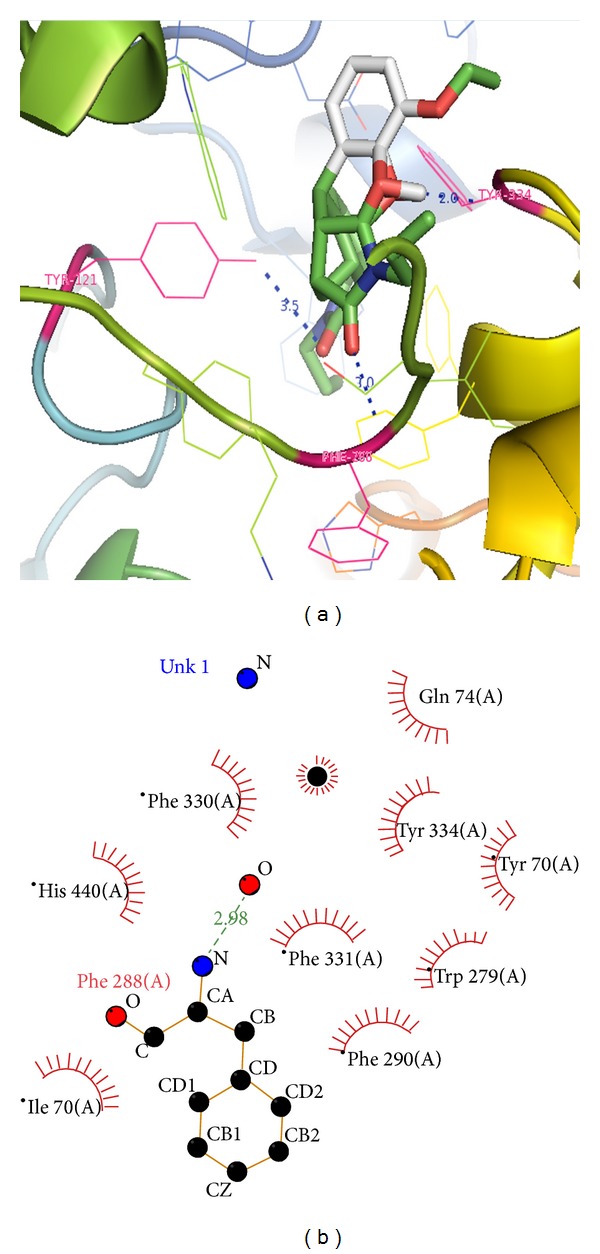
Docking and Ligplot interaction of AChE/CID: 1990283 (C_28_H_34_N_2_O_6_) before simulation, (a) Docked ligand in active binding pocket, (b) 2D diagram of ligand and protein residue contacts.

Among 500 herbal lead compounds top 10 ligands were subsequently analyzed for binding pattern with AChE using AutoDock methods on the basis of binding energy score and further analyzed for possible molecular interactions with AChE using LigPlot program and visualized by Python Molecular Viewer (PyMol) (Table 1) and as per our previous studies [33].* Cannabis* plant's secondary metabolite C_28_H_34_N_2_O_6_ (CID: 1990283) was found interacting with active site residue Phe288 of AChE through one hydrogen bond with 2.98 Å and nine hydrophobic interactions, obeying Lipinski's rule of five, having lowest minimum binding energy of −12.61 Kcal/Mol, log⁡⁡*P* = 2.91, inhibition constant (*K*_*i*_) = 570.38 nM, and total intermolecular energy of −13.49 Kcal/Mol while second rank derivative (CID: 1991460) was interacting through two hydrogen bonds and eight hydrophobic interactions with active site residues Phe288 and Asp121, obeying Lipinski's Rule as well, having second lowest minimum energy of −11.18 Kcal/Mol, log⁡⁡*P* value = 1.24, inhibition constant (*K*_*i*_) = 6.40 nM, and total intermolecular energy of −11.75 Kcal/Mol (Figure 1 and Table 1). On the basis of complex scoring and interactions with the active site residue and binding ability it was deciphered that THC derivative specifically CID: 1990283 could be promising inhibitor of AChE.

**Figure 2:**
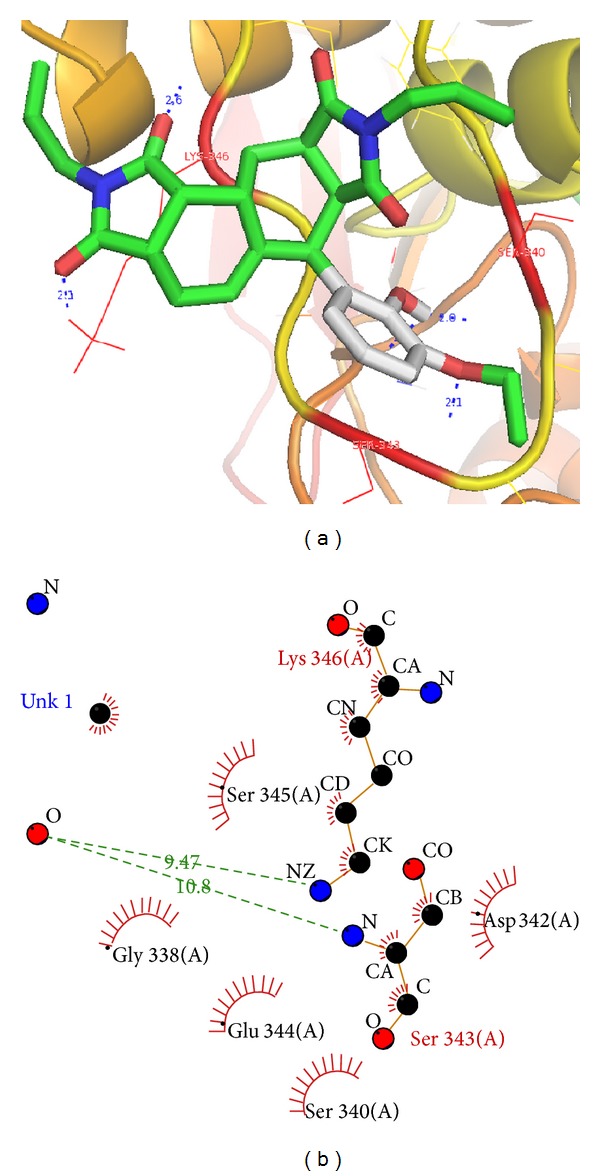
Docking and Ligplot interaction of AChE/CID: 1990283 (C_28_H_34_N_2_O_6_) after simulation, (a) Docked ligand in active binding pocket, (b) 2D diagram of ligand and protein residue contacts.

**Figure 3:**
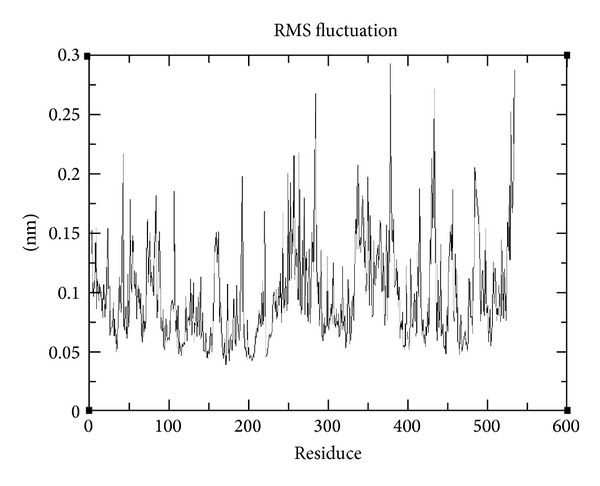
Root mean square fluctuations in AChE/CID: 1990283 (C_28_H_34_N_2_O_6_) during simulation.

Since molecular docking provides only a static view of protein-ligand interaction, hence we also performed molecular dynamics simulation on ChE/C28H34N2O6 complexes in order to study the interactions in motion, fluctuation in residues, and movement in any specific domain or separation of protein ligand complex. The overall goal of this simulation step was to account for protein flexibility and movement that cannot be achieved in the docking simulation also to check the stability of the complex interactions [34].  Figure 2 shows the interactions of the docked C_28_H_34_N_2_O_6_ with AChE in a 1000 ps simulated snapshot. Two hydrogen bonds with Ser343 and Lys346 with 5 hydrophobic bond interactions have been formed during course of simulation in water (Table 2 and Figure 2). On careful observation by aligning the two docked structures, the presimulated one with the 1000 ps simulated snapshot, it was found that the ligand substantially moves towards these residues to form additional H-bonds. Moreover, it was observed that the ligand was slide down into the deep gorge cavity to stabilize contacts with active site residues and to avoid further fluctuations in structural confirmations.

**Figure 4:**
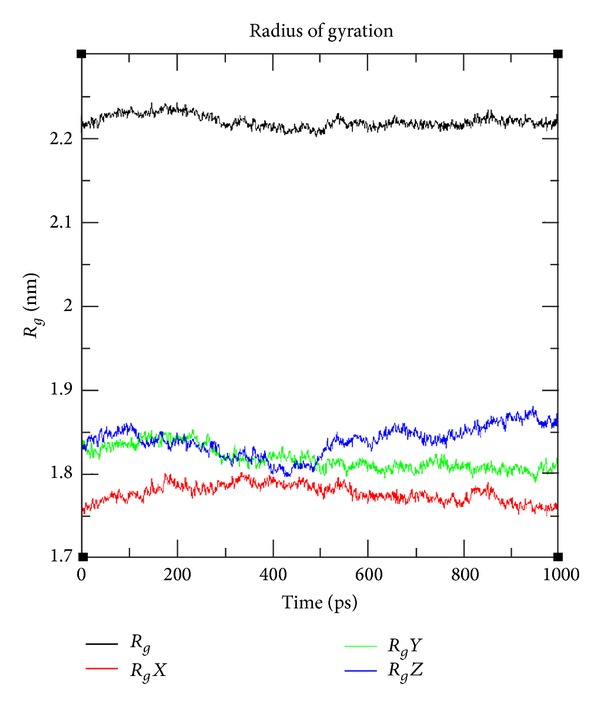
Radius of gyration of AChE/CID: 1990283 (C_28_H_34_N_2_O_6_).

To examine in detail the ligand-receptor interactions of AChE/C_28_H_34_N_2_O_6_ complex and to estimate the dynamic stabilities of the hydrogen bonds facilitating the inhibitor in the active site of AChE, we calculated the time evolutions of the associated interatomic distances and contacts. For this complex, two hydrogen bonds with Ser343 and Lys346 and 5 hydrophobic were observed, with percentage occupancy over 50 for the entire simulation. The minimum binding energy was decreased from −12.61 to −8.64 Kcal/Mol but more importantly two hydrogen bonds were found instead of one (Figures 1 and 2). We also calculated RMSDs between C*α* of AChE trajectories recorded every 1000 ps and C*α* of their X-ray crystal structure. The RMSDs for the trajectory of AChE in complex with C_28_H_34_N_2_O_6_ were also calculated using its initial docked structure as a reference. The simulation length used in the entire study was long enough to allow rearrangement of side chains of the native as well as the drug complex protein thus facilitating the most stable binding mode. The folding of biological macromolecules is among the main problems of molecular biology and biophysics although having a huge set of possible conformations; a protein assumes its unique stable spatial structure within a time. The molecular spatial packing of amino acid residues is an important aspect of protein stability, which was evaluated by calculating radius of gyration. The higher the value of gyration is, the less is the compactness [34]. At aqueous environment condition gyration value reaches 0.40 nm for AChE/C_28_H_34_N_2_O_6_ complex (Figure 4). Hence, AChE structure was found to be less compact and more accessibility of binding pocket residues to our ligand C_28_H_34_N_2_O_6_. The greater degree of movement has been observed in the AChE, when simulated under aqueous condition.

Based on the results from AChE/C28H34N2O6 complex it appears that molecular interactions of C28H34N2O6 with the residues Ser343, Ser340, and Lys346 in AChE are important for inhibitory activity. A comparison between the conformations obtained from docking and molecular dynamics simulations showed substantial changes in binding conformations. These results indicate initial receptor-ligand interaction observed in docking can be limited due to the receptor rigid docking algorithm but the conformational changes and interactions observed after simulation runs are more energetically favoured and better representations of derivative poses in receptor.

## 4. Conclusion

We have performed docking and molecular dynamics simulation studies to elucidate the binding mechanism of prospective herbal drug C_28_H_34_N_2_O_6_ into the structure of AChE. Further, long simulations for 1 ns suggest that ligand interactions with the residues Asp72, Tyr70-121-334, and Phe288 of AChE, all of which fall active site/subsite or binding pocket, may be critical for its inhibitory activity. The present MD simulations support the hypothesis that C_28_H_34_N_2_O_6_ is quite a probable and a worth small molecule ligand for targeting/inhibiting the acetylcholinesterases. These results would be valuable for further designing noncovalent type inhibitors with high specificity and potent activity.

## Figures and Tables

**Table 1:** Top ten THC derivative inhibitors of ACHE identified from ChemBank database after virtual screening.

CID	Binding energy (Kcal/mol)	MW (g/mol)	H-Bond interaction	Hydrophobic interaction	log⁡*P*	Estimated inhibition constant, (*K*_*i*_) nM	Total intermolecular interaction energy, Kcal/Mol
1990283	−12.61	494.58	1	9	2.91	570.38	−13.49
1991460	−11.18	466.53	2	8	1.24	6.40	−11.75
1377639	−9.82	567.67	1	6	5.80	63.54	−11.56
1986809	−9.72	359.42	1	7	2.14	75.42	−10.45
1990059	−9.66	420.42	4	7	6.40	82.52	−10.68
1990307	−9.65	410.42	2	6	0.75	84.55	−10.78
1989979	−9.53	392.37	3	6	5.54	103.88	−10.14
3076287	−9.44	354.40	1	9	4.40	120.47	−10.85
1620276	−9.34	475.58	1	10	4.43	142.74	−11.20
3553198	−9.28	408.49	4	6	6.08	56.62	−11.45

**Table 2:** THC derivative inhibitors of ACHE after molecular dynamics simulation.

Complex	Energy score (kcal/mol)	Number of H-bond interaction	Hydrophobic interaction	Total intermolecular interaction energy, Kcal/Mol	Estimated inhibition constant, (*K*_*i*_) nM
2W9I/CID: 1990283	−6.01	2 (Ser343, Lys346)	5	−8.64	570.38
